# Enhanced Estimation of Root Zone Soil Moisture at 1 km Resolution Using SMAR Model and MODIS-Based Downscaled AMSR2 Soil Moisture Data

**DOI:** 10.3390/s21155211

**Published:** 2021-07-31

**Authors:** Maedeh Farokhi, Farid Faridani, Rosa Lasaponara, Hossein Ansari, Alireza Faridhosseini

**Affiliations:** 1 Department of Water Science and Engineering, Ferdwosi University of Mashhad, Mashhad 9177948974, Iran; maedeh.farokhi@mail.um.ac.ir (M.F.); ansary@ferdowsi.um.ac.ir (H.A.); farid-h@ferdowsi.um.ac.ir (A.F.); 2 DICEM, Department of European and Mediterranean Cultures, Environment, and Cultural Heritage, University of Basilicata, 75100 Matera, Italy; 3 Institute of Methodologies for Environmental Analysis, National Research Council (CNR), 85050 Tito Scalo, Italy

**Keywords:** downscaling, AMSR2, MODIS, SMAR, soil moisture

## Abstract

Root zone soil moisture (RZSM) is an essential variable for weather and hydrological prediction models. Satellite-based microwave observations have been frequently utilized for the estimation of surface soil moisture (SSM) at various spatio-temporal resolutions. Moreover, previous studies have shown that satellite-based SSM products, coupled with the soil moisture analytical relationship (SMAR) can estimate RZSM variations. However, satellite-based SSM products are of low-resolution, rendering the application of the above-mentioned approach for local and pointwise applications problematic. This study initially attempted to estimate SSM at a finer resolution (1 km) using a downscaling technique based on a linear equation between AMSR2 SM data (25 km) with three MODIS parameters (NDVI, LST, and Albedo); then used the downscaled SSM in the SMAR model to monitor the RZSM for Rafsanjan Plain (RP), Iran. The performance of the proposed method was evaluated by measuring the soil moisture profile at ten stations in RP. The results of this study revealed that the downscaled AMSR2 SM data had a higher accuracy in relation to the ground-based SSM data in terms of MAE (↓0.021), RMSE (↓0.02), and R (↑0.199) metrics. Moreover, the SMAR model was run using three different SSM input data with different spatial resolution: (a) ground-based SSM, (b) conventional AMSR2, and (c) downscaled AMSR2 products. The results showed that while the SMAR model itself was capable of estimating RZSM from the variation of ground-based SSM data, its performance increased when using downscaled SSM data suggesting the potential benefits of proposed method in different hydrological applications.

## 1. Introduction

Spatiotemporal distribution of root zone soil moisture (RZSM) across massive areas of land significantly contributes to lots of meteorological, hydrological, and agricultural applications [1,2]. Moreover, RZSM estimation at different spatiotemporal scales has a substantial role in strategic water resources management [3]. RZSM is a key storage parameter governing mass and energy partitioning associated with runoff and evapotranspiration (ET) [4]. The temporal evolution and spatial distribution of RZSM are affected by land-use, precipitation, soil texture, topography, and various meteorological variables [3].

Having gathered in the field, soil moisture (SM) measurements—typically known as in situ measurements—can be generally retrieved from low- or high-density networks of point measurements. To determine network density, the amount and assembly of tools, project budget, and study area should be taken into account [5]. Pointwise-scale SM measurements are conducted using ground-based SM methods. These measurements fail to demonstrate SM values for adjacent areas due to the large SM spatial heterogeneity on a vast array of scales (e.g., [6,7,8]). Likewise, large-scale ground-based SSM extraction through geostatistical methods is not suitable due to the large spatial heterogeneity. On the other hand, there are yet no dense SM-monitoring networks in many large areas. Therefore, SM measurement on different scales with ground-based instruments is still challenging.

Recent technological and theoretical advancements have paved the way for using remote sensing techniques in measuring SM content. Active and passive microwave observations have been widely used to estimate SM (e.g., [9,10,11]) and the SSM products are freely available for many applications. Table 1 summarizes the specifications of some of the sensors providing remotely sensed SM products [12,13].

Satellite-derived remotely sensed SSM products have their own advantages, including the accessibility of global-scale measurements at a continuous spatiotemporal resolution (STRs). Nevertheless, satellite-based products are currently accessible at low spatial resolutions, rendering them useless for small-scale agricultural applications and hydrologic models [14,15]. The satellite-derived product validation process is severely restricted in the presence of ground-based networks. Recently, research has focused on improving the estimated SSM of satellite-derived products by applying various algorithms connecting SSM with variables like brightness temperature, precipitation, vegetation, and so on [2,15,16,17,18]. The majority of techniques applied to derive high-resolution SM from synergies between microwave and optical observations rest upon triangular and trapezoidal approaches. Refs. [18,19] proposed an empirical polynomial fitting downscaling approach based on the frequently used triangular feature space formed by surface temperature (Ts) and vegetation index (VI). Here, high spatial resolutions are expressed as a polynomial function of land-surface temperature (LST), Normalized Difference of Vegetation Index (NDVI), and opticothermal data-derived surface albedo as a region/climate conditions-specific regression formula. The polynomial fitting downscaling approach was employed to downscale SM and ocean salinity (SMOS), AMSR-E SM with high-resolution surface variables from Meteosat second-generation spinning enhanced visible and infrared imager (MSG-SEVIRI) or moderate resolution imaging spectroradiometer (MODIS) observations (see, for example, [20,21,22,23,24]).

Microwave sensor-derived SM data are immediately associated with the surface soil layer (0.2 cm to 5 cm) [25,26]. However, the largest part of the energy and water budgets of an ecosystem depends heavily on the spatial distribution of RZSM. Examining the correlation between SSM and RZSM in various studies reveal that RZSM is a function of SSM [27,28,29]. It has been particularly challenging to delineate the analytical relationships between SM and SSM in lower soil layers (see, for example, [30,31,32,33]), requiring further investigation. Studies have been conducted on how RZSM is estimated from SSM [34,35,36,37]. The presented methods involve simple statistical relationships with physically based methods. They require a series of experimental parameters alterable with soil type and vegetation. Here, soil profile moisture is estimated by assuming the conditions of a hydraulic balance between momentary SSM and soil profile moisture [37,38].

By solving a simple water balance equation for arid and semi-arid zones, ref. [37] developed a soil moisture analytical relationship (SMAR) model to express the relationship between RZSM and SSM. The results of implementing the SMAR model on measuring SM at different depths indicated that this model could predict RZSM on both local and regional scales [29,37,39]. SSM is one of the main parameters in the SMAR model, which can use satellite-based SSM data to estimate RZSM [29,39]. By applying remotely sensed SSM TRMM-TMI products (with coarse resolution 25 km), ref. [39] evaluated the estimated RZSM using the SMAR model at several stations in North America. Although satellite-based SSM products represent large-area spatial mean and assign the data to a specific point therein, it could not be effective despite being successful in some cases. They proposed in their study to increase the resolution using downscaling techniques [39].

The spatiotemporal accessibility and accuracy of SSM and RZSM measurements are significant components to obtain optimal results in a variety of applications. There are also extremely restricted resources accessible to reclaim accurate SM measurements in the Rafsanjan plain (RP). Thus, this study will assess AMSR2 data quality using REC-P55 SM sensors manufactured by Ansari and Hassanpour [40] and apply a downscaling approach to better characterize satellite-derived SSM when traveling above the study area (Rafsanjan Plain). The performance of this method will be evaluated based on the differences between ground-based and satellite-derived SSM measurements. The downscaling method used consists of a simple linear equation that correlates AMSR2 SM with three parameters retrieved from MODIS: Albedo, LST, and normalized difference vegetation index (NDVI). Afterwards, to estimate RZSM, the downscaled SSM values will be utilized in the SMAR model and the results will be compared with the measured data. At the end, it can be said that the purpose of this study is to provide a methodology to implement the downscaled SSM values to prepare soil profile maps at 1-km resolution in a study area.

At the end, a summary of the established and emerging soil moisture retrieval methods as well as the scientific challenges seem necessary to understand the milestones in this field of study. According to a latest review, the state-of-the-art topics can be summarized into five items [2]:

1. VIS/NIR, TIR, and microwave are currently the three main data sources for global SSM monitoring. The VIS/NIR and TIR data have higher spatial resolution and can easily be affected by clouds, while microwave data have lower spatial resolution and can provide all-weather data coverage. To overcome the gaps between these sources of data, the synergic multi-band SSM retrievals should be considered.
2. Considerable efforts have been made in the retrieval of two Land Surface Temperature (LST) and SSM using passive microwave observations and auxiliary data. However, SSM and LST are codependent making the retrieval process difficult. Moreover, obtaining accurate auxiliary information such as meteorological parameters and soil texture is difficult due to clear-sky dependency and low spatial resolution, respectively. To solve this problem, it is essential to simultaneously retrieve LST and SSM from only passive microwave data to reduce the number of unknown parameters and make the retrieval independent of auxiliary information.
3. The target accuracy of RMSE of 0.04 m^3^/m^3^ between the satellite and measured SSM data has been the criterion for validating the SSM products, however, scientists suggested that distinguished target accuracies should be determined for different combination of surface moisture status, soil texture, and vegetation coverage.
4. In order to validate remotely sensed SSM data, in situ SSM measurements with a fixed depth of 5 cm are frequently used. However, VIS/NIR and microwave data can reflect SSM with only a few millimeters and centimeters, respectively, mostly depending on SSM content within the soil column and frequencies used for detecting SSM. Therefore, it seems that currently there is no good way to solve the contradiction of sensing depth, however, post-processing steps (e.g., assimilation technology and the entropy theory) can mitigate this problem.
5. The estimation of RZSM from satellite SSM has been increasingly proposed by a number of investigations. The possible approaches for obtaining such data are (a) assimilating microwave SSM data into land surface models; (b) exponential filter and the analytical relationship between RZSM and SSM; and (c) using the P-band SAR with much deeper penetration depth due to its longer wavelength. However, satellite SSM accuracy and soil texture distribution are among the great challenges to obtain RZSM at present.

## 2. Materials and Methods

### 2.1. Study Area

**Figure 1:**
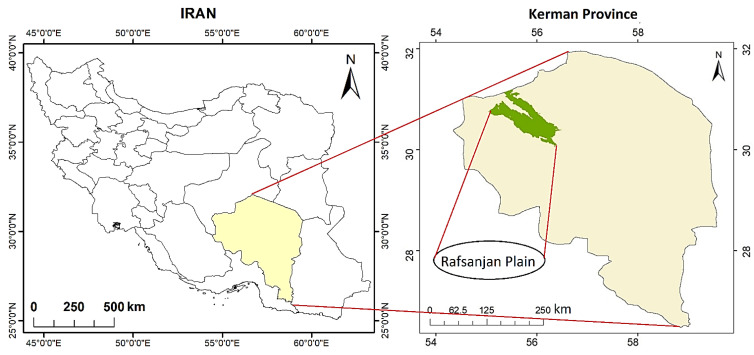
Geographical location of Rafsanjan Plain within Iran and Kerman Province.

In this study, the Rafsanjan Plain was taken as the area of study, situated in southeast Iran, Kerman Province (Figure 1), Iran’s center of pistachio cultivation, comprising a total area of 5622 km^2^. The study area is located between 55°1′ to 56°28′ longitude and 30°3′ to 31°11′ latitude, where the elevation ranges from 1296 to 2131 m above sea level (ASL). The average annual rainfall and average annual potential evapotranspiration (PET) here are below 100 mm and above 3000 mm, respectively. There is a thermic soil temperature regime and an aridic SM regime here.

### 2.2. Data

#### 2.2.1. AMSR2 Satellite-Based SSM Data

The Global Change Observation Mission–Water 1 (GCOM-W1) satellite system was launched on 17 May 2012, to gather geophysical parameters, such as precipitation, sea surface temperature (SST), and SM, and witness variation in water circulation [41]. It carries the AMSR2 sensor aimed at retrieving earth-emitted radiometric waves and data utilized to estimate global-scale low-resolution SSM with a medial temporal resolution of a couple of days [41,42]. The land parameter retrieval model (LPRM) is extensively used to estimate SSM based on a radiative transfer forward (RTF) model responsible for the retrieval of vegetation optical depth (VOD) and SM. One can refer to the Japan aerospace exploration agency (JAXA) website (data downloadable in HDF5) or the NASA website (data downloadable in netCDF4), scenes (all measurements taken halfway between Earth’s North and South Poles relative to observation point) or a global map (10 km and 25 km resolution), daily or monthly, to retrieve AMSR2 SSM products from Earth observation research center (EORC) for day/night readings.

This study retrieved and analyzed the data of daily global AMSR2 SSM estimates at 25 km spatial resolution from 19 July 2017 to 30 August 2018 for ascending (Asc.) overpasses. AMSR2 SSM data at 25 km spatial resolution were only available for Rafsanjan area by employing its geographic coordinates (https://hydro1.gesdisc.eosdis.nasa.gov/) (accessed on 18 April 2018). The complete ID of the product is: AMSR2/GCOM-W1 surface soil moisture (LPRM) L3 1 day 25 km × 25 km ascending V001 (LPRM_AMSR2_A_SOILM3) [43].

#### 2.2.2. Ground-Based SM Data

**Figure 2:**
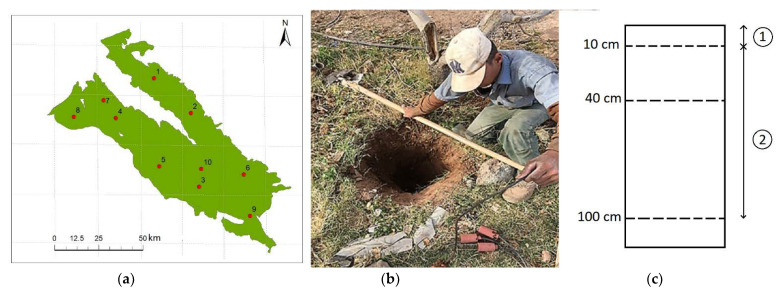
(**a**) Location of stations in Rafsanjan plain, (**b**) REC-P55 SM sensors manufactured by Ansari and Hassanpour [40] installed in study wells at three different depths of 10, 40, and 100 cm, and (**c**) two layers of surface and deep (root zone) soil considered in the SMAR model.

To obtain ground-based measured SSM and RZSM data in the Rafsanjan Plain, REC-P55 SM sensors (presented by [40]) were installed in ten locations (Figure 2a) at three different depths of 10, 40, and 100 cm (Figure 2b). The daily soil temperature and moisture voltage were measured and recorded at 01:30pm (approximate ascending satellite overpass time) by a data logger. Sensors were pre-calibrated, and the moisture equations of each soil type were obtained for sensors by utilizing the weighing technique in the laboratory.

This study primarily analyzed SSM in each location during satellite crossing, followed by the analysis of soil profile moisture data. Two layers of surface and deep (root zone) soil with 10 and 90 cm depths were considered in the SMAR model, respectively (Figure 2c). RZSM (10–100 cm depth) was equivalent to the weighted average measured SM by measuring sensors at depths of 10, 40, and 100 cm at selected stations of the study area. SSM (0–10 cm depth) was also measured by the measurement sensor in the surface layer.

#### 2.2.3. Remotely Sensed MODIS Parameters

**Figure 3:**
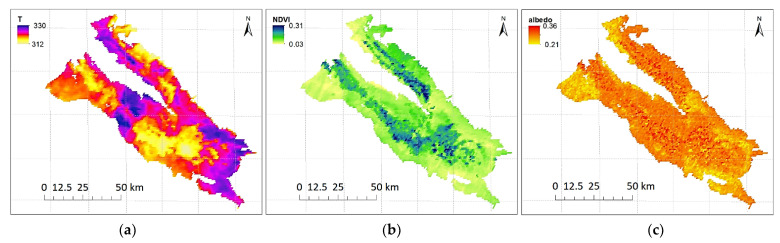
MODIS (**a**) land surface temperature, (**b**) NDVI, and (**c**) Albedo products of Rafsanjan Plain for random days during the study period.

The MODIS sensor was launched on board the Terra satellite in December 1999 and the Aqua satellite in May 2002, both belonging to NASA’s international Earth Observing System (EOS), for data collection. They both revolve in a circular heliosynchronous orbit, enabling them to circle the earth every 99 min, i.e., 16 orbits each day, and gather information for the whole planet every one or two days. MODIS Albedo, LST, and NDVI products at a 1 km spatial resolution were retrieved in HDF4 format from EOS data and information system (EOSDIS) website (https://search.earthdata.nasa.gov/) (accessed on 13 April 2018) for the area of study. For this research, MODIS products, including MYD11A1 (Aqua Satellite), were used to estimate 1 km resolution LST (Figure 3a), and 16-day dataset MOD13A2 (Terra Satellite) and MYD13A2 (Aqua Satellite) were used to create 8-day NDVI layers (Figure 3b). Surface albedo(α) was also estimated from a combination of bands 1 to 7 of the MODIS product (MOD09A1) (Figure 3c).

### 2.3. AMSR2 SSM Downscaling

The SSM data are one of the most important input parameters to be used in the RZSM estimation model, though it is affected by several factors, such as daily rainfall, impermeable areas, land-use, soil temperature and type, and vegetation density, introducing high variability even in small scales. Thus, any coarse resolution SSM data (>1 km) is not expected to represent variability of this parameter in small-scale application. Downscaling generally aims to set up either a physically based model or a statistical correlation between fine-scale auxiliary variables and coarse-scale SM [44]. Previous studies proved that there is a correlation between the SSM, topographic/vegetation characteristics, and soil properties [45,46]. This information is applicable during downscaling because the assessment of the relationships between SSM and for example, soil texture and topography, typically demands comprehensive observations. The approach below was adopted to enhance satellite-derived continuous spatiotemporal SSM measurements over the Rafsanjan Plain. The downscaling method used here was initially released by [19] and has been implemented formerly by [47]. Based on this method, remotely sensed SSM retrieved from AMSR2 at a 25 km spatial resolution could be downscaled to a 1 km spatial resolution by utilizing a simple linear equation in accordance with parameters estimated using a regression model founded on three physical properties (i.e., albedo, LST, and NDVI) at a MODIS-retrieved 1 km spatial resolution. The downscaling technique with respect to the above-mentioned triple parameters is expressed by Equation (1) [19]:

(1) $$\theta_{s}=\overset{i=n}{\underset{i=0}{\sum }}\overset{j=n}{\underset{j=0}{\sum }}\overset{k=n}{\underset{k=0}{\sum }}a_{ijk}V^{i}T^{j}A^{k}$$

Setting the number of explanatory variables to 1 in Equation (1) yield:

(2) $$\theta_{s}=a_{000}+a_{100}V+a_{010}T+a_{001}A+a_{110}VT+a_{101}VA+a_{011}TA\;$$

where a_ijk_ parameters represent the correlation between satellite-derived coarse-resolution SSM product and fine-resolution downscaled SSM product. They can be measured using the multiple linear regression (MLR) model, comparing coarse-resolution SSM estimates and aggregate values of physical properties. A, T, and V represent albedo, LST, and NDVI, respectively. Each of the parameters was up-scaled to correspond toAMSR2 at a 25 km spatial resolution as follows [19]:

(3) $$V_{25km}=\frac{\sum_{i=1}^{n}\sum_{j=1}^{m}V_{i,j}}{mn}\;;T_{25km}=\frac{\sum_{i=1}^{n}\sum_{j=1}^{m}T_{i,j}}{mn}\;;\;A_{25km}=\frac{\sum_{i=1}^{n}\sum_{j=1}^{m}A_{i,j}}{mn}\;$$

where 25 km denotes the resolution at which upscaling of physical parameters is performed, m denotes the value for the *i*th column of the grid with a 1 km spatial resolution within the 25 km spatial resolution and n denotes the value for the *j*th row of the grid with a 1 km spatial resolution within the 25 km spatial resolution.

### 2.4. SMAR Model

Based on soil physical properties, the soil moisture analytical relationship (SMAR) model correlates between SSM and RZSM. SMAR assumes two layers for the soil: (1) a surface layer (a few centimeters deep, seen as equivalent to retrieval depth of satellite-based SSM product), and (2) a layer below the surface layer extending to the vegetation rooting depth [37]. The only water mass exchange between the aforesaid layers is infiltration, with other processes, including the capillary rise and lateral flow, assumed insignificant. Equation (4) was proposed by [37], describing the instantaneous infiltration flux from the upper to the bottom layer.

(4) $$n_{1}{\mathrm{Lr}}_{1}I\left(t\right)=n_{1}{\mathrm{Lr}}_{1}I\left[s_{1}\left(t\right),t\right]=n_{1}{\mathrm{Lr}}_{1}\{\begin{array}{c} \left(s_{1}\left(t\right)-s_{c1}\right),s_{1}\;\left(t\right)\geq s_{c1}\\ \;0\;,\;s_{1}\;\left(t\right)\geq s_{c1} \end{array}$$

In the above equation, I (t) [[–] represents the amount of saturated soil penetrating the lower layer, *n_1_* [[–] represents top soil porosity, *Lr1* (L) represents top soil depth, $s_{1}\left(\frac{\theta_{\mathrm{top}}}{n_{1}}\right)\left[-\right]$ represents relative saturation of soil in the first layer, and sc1 represents relative saturation at topsoil field capacity. SSM needs to be taken as referred to the first 5 to 10 cm of soil. Even though the majority of satellite sensors fail to see deeper than a few centimeters, it can be reasonably assumed that these measures can represent the dynamics of a ≈ 5–10 cm surface layer [37]. The depth of surface layer in the SMAR model should not be less than 5 cm, otherwise the model might underestimate the infiltration and face numerical problems. While most satellite sensors provide information about soil water content no deeper than 0–5 cm of the top soil, a number of studies proved that satellite products can capture the required SSM dynamics for the surface layer of SMAR model [29,39,48,49,50].

By defining $x_{2}=\left(s_{2}-s_{w2}\right)/\left(1-s_{w2}\right)$ as the effective relative saturation at second layer of soil and $\omega_{0}=(1-s_{w2})n_{2}Z_{r2}$ as the soil water storage, the SMAR soil water balance is described as:

(5) $$\left(1-s_{w2}\right)n_{2}Zr_{2}\;\frac{dx_{2}\left(t\right)}{dt}=n_{1}Zr_{1}y\left(t\right)-V_{2}x_{2}\left(t\right)$$

where $s_{2}$ [[–] is current relative saturation, $s_{w2}$ [[–] relative saturation at wilting point, n_2_ [[–] soil porosity, $Zr_{2}$ [L] second layer depth, $V_{2}$ [${L\;T}^{-1}$] soil water loss coefficient (both evapotranspiration and percolation losses), and $x_{2}$ [[–] effective relative soil saturation of the second soil layer. The second term of the right side of the Equation (5) represents a linear soil water loss function where soil water loss would be linearly reduced from a maximum value at the saturation point to zero at the wilting point. We can simplify Equation (5) by standardized coefficients:

(6) $$a=\frac{V_{2}}{\left(1-s_{w2}\right)n_{2}{\mathrm{Lr}}_{2}}\;,\;b=\frac{n_{1}{\mathrm{Lr}}_{1}}{\left(1-s_{w2}\right)n_{2}{\mathrm{Lr}}_{2}}$$

where $s_{w2}\left[-\right]$ denotes relative saturation of soil in the second layer at wilting point (WP), $n_{2}\left[-\right]$ denotes porosity of the soil in the second layer, ${\mathrm{Lr}}_{2}\left[-\right]$ denotes the depth of soil in the second layer and $V_{2}\left[{\mathrm{LT}}^{-1}\right]$ denotes SM loss coefficient justifying percolation as well as ET losses. Finally, $s_{2}\left(\theta_{\mathrm{sub}}/n_{2}\right)\left[-\right]$ represents relative saturation of the second soil layer, which can be measured using the following equation [37]:

(7) $$s_{2}\left(t_{j}\right)=s_{w2}+\left(s_{2}\left(t_{j-1}\right)-s_{w2}\right)e^{-a\left(t_{j}-t_{j-1}\right)}+\left(1-s_{w2}\right)\mathrm{bI}\left(t_{j}\right)\left(t_{j}-t_{j-1}\right)$$

The SMAR parameters for different soil textures are shown in Table 2 [37,51]. In this study, the daily RZSM (10–100 cm depth) was equivalent to the weighted average measured SM by measurement sensors at depths of 10, 40, and 100 cm at selected stations of the study area. SSM (0–10 cm depth) was also measured by the measurement sensor in the surface layer. The depth of surface layer in the SMAR model should not be less than 5 cm, otherwise the model might under-estimate the infiltration and face numerical problems [39]. While most satellite sensors provide information about soil water content no deeper than 0–5 cm of the top soil, a number of studies demonstrated that satellite products can capture the SSM dynamics required for the SMAR model surface layer [29,39,44,45,46].

Three input sources were envisaged for the SMAR model (three schemes) in order to investigate the effect of spatial resolution of the results: (1) the SMAR model based on ground-based measured SSM, which estimates RZSM values at selected stations, (2) the SMAR model based on AMSR2 25 km SSM, and finally (3) the SMAR model based on downscaled 1 km SSM data using MODIS products.

## 3. Results

### 3.1. AMSR2 Downscaling Using MODIS Albedo, LST and NDVI

**Figure 4:**
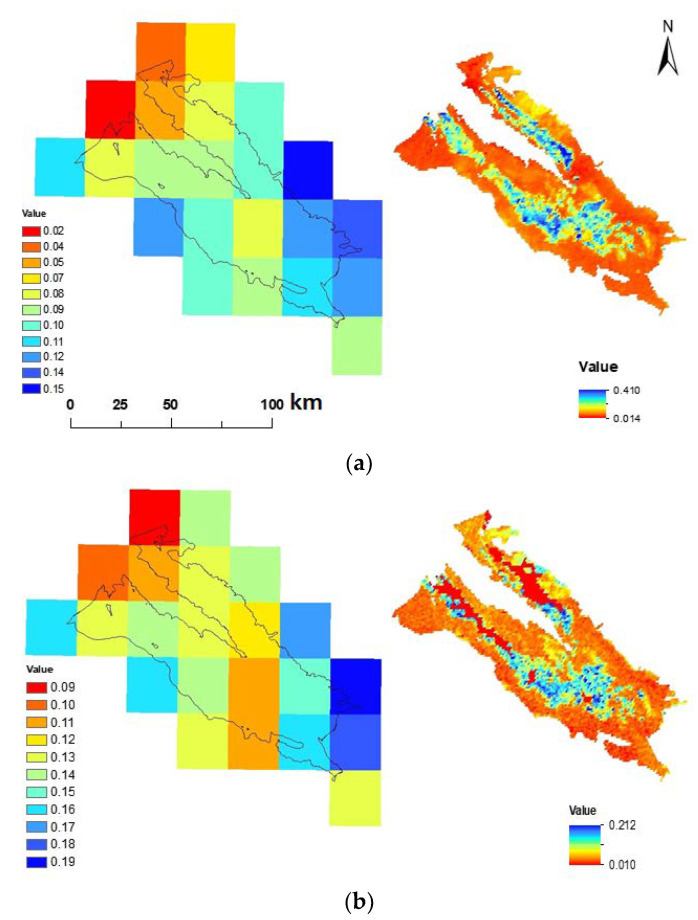

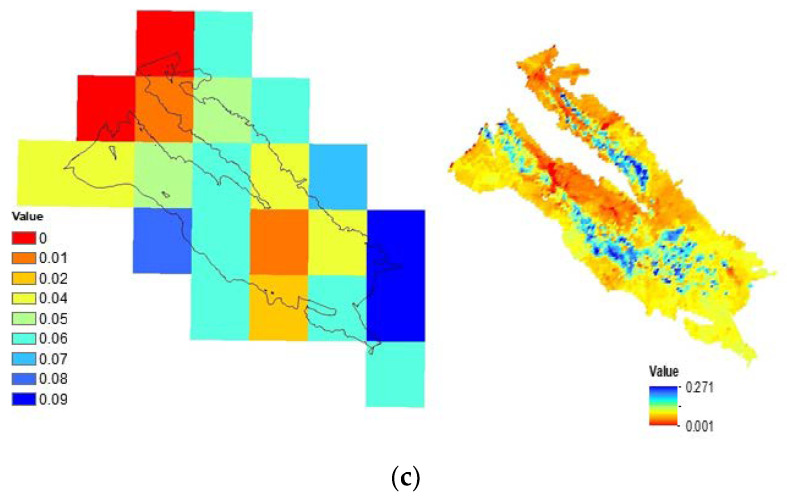
The conventional AMSRE (Left) and downscaled AMSRE (Right) SSM maps of the Rafsanjan plain on (**a**) 19-09-2017, (**b**) 01-01-2018, (**c**) 08-07-2018, and (**d**) 8/29/2018.

The AMSR2 SSM product at a 25 km spatial resolution was downscaled to 1 km spatial resolution using a simple linear equation containing MODIS Albedo, LST, and NDVI products (Figure 4). The following equation shows how AMSR2 data are downscaled:

θ_s_ = 0.893 − 0.931 A − 0.256 V − 0.0025 T + 0.0027 TV − 0.00226 TA − 2.133 VA (8)

In the above equation, θs represents downscaled SSM. A, T, and V represent albedo, LST, and NDVI, respectively. The remainder is A-T-V interactions (i.e., TA, VA, and VT). The regression model was obtained with R^2^ = 0.61 and the acceptable *p*-value (<0.05) and F-statistic test (=$4.3\times 10^{-248}$).

Table 3 shows that *p*-values are below the significance level of 5%, meaning that the null hypothesis for each coefficient is rejected and all components are significant in the linear equation. The results indicated that the downscaling model offered an excellent fit with the AMSR2 estimates.

The AMSR2 25 km SSM and downscaled SSM values were examined by calculating MAE, RMSE, and R for every station. According to Table 4, comparing AMSR2 25 km SSM and measured values in every station revealed that the results obtained were not satisfactory for most of the stations. The highest MAE and RMSE values were obtained at Station 2. The R-value was also estimated as low at Stations 1, 2, 3, 6, and 7, which could be affected by the distance from the station to the centroid (distance) at each AMSR2 25 kmpixel, such that R values were found to be acceptable at Stations 4, 5, and 10.

**Figure 5:**
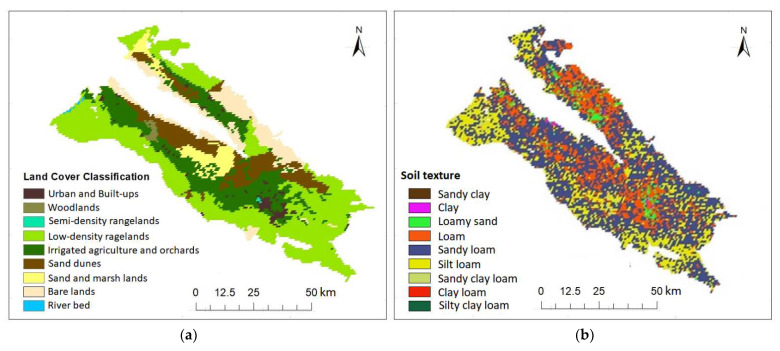
(**a**) Landuse/landcover map, and (**b**) soil texture map of the Rafsanjan Plain.

Other factors such as changes in vegetation density, land cover, land use, etc., at AMSR2 25 km pixel could also affect the results of MAE, RMSE, and R. Evaluating the land cover map of the study area (Figure 5a) showed that vegetation was uniform at 25 km pixels containing Stations 4, 5, 8, 9, and 10 (dominant vegetation at 25 km × 25 km pixels containing Stations 4, 5, and 10 includes irrigated agriculture and pistachio orchards, and at pixels containing Stations 8 and 9, it includes rangeland). These stations were located at places similar to the dominant vegetation cover (VC) in the pixel in terms of vegetation, influencing the superiority of results obtained from there. However, VC at Station 2 was different from the dominant vegetation of the area located in the pixel (dominant cropping pattern in this pixel included low-density rangelands and non-vegetated lands). This led to increased MAE and RMSE values and decreased R-value, save for the distance. Thus, it can be stated that the AMSR2 25 km SSM values exhibit a large-area spatial mean (25 km). Assigning the data to all points in there was ineffective, despite being successful in some stations. Hence, the area must be reduced using downscaling methods.

**Figure 6:**
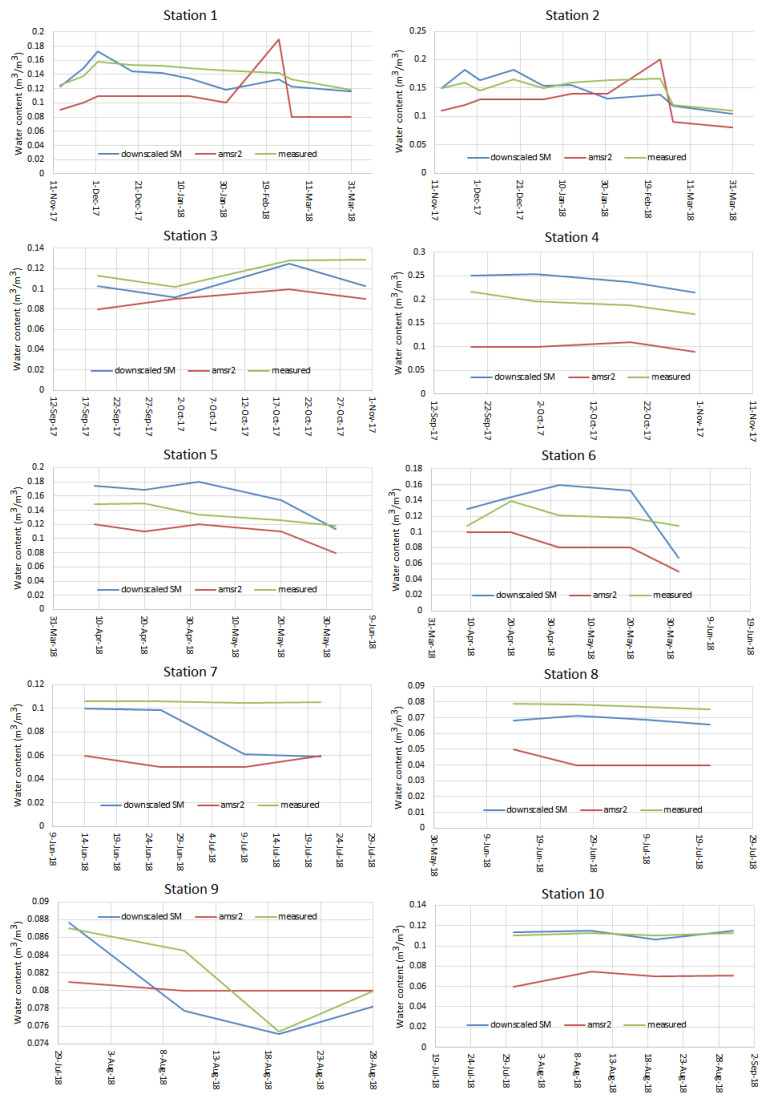
Time series of Measured, ordinary AMSR2, and downscaled ASMR2 SSM data at ten study stations. Stations 1–10 correspond to the numbered red dots in Figure 1.

According to Table 4, comparing downscaled SSM results with the measured values at each station indicated a decreased error rate, increased correlation coefficient, and improved SSM results for the stations far from the midpoint of the pixel (Figure 2a), all mediated by the downscaling technique used. Consequently, at Stations 1, 2, 3, and 7, error values decreased, and correlation coefficients increased. Therefore, the downscaling method that uses MODIS parameters was arguably more correlated with measured SSM data. The time series of Measured, ordinary AMSR2, and downscaled ASMR2 SSM data at 10 study station has been depicted in Figure 6 as well. The increase in the consistency and accuracy of the downscaled AMSR2 data with respect to the measured SSM data in almost all stations is visible in Figure 6.

### 3.2. RZSM Estimation Based on the SMAR Model

Three of the four SMAR parameters (i.e., n, sw, and sc) were determined based on soil texture map of the study area (Figure 5b), according to Table 2. Considering the SM loss coefficient justifying percolation and ET losses (V_2_) in the SMAR model, the V_2_ value was obtained 5.8 mm/day regarding optimization based on regional conditions using the genetic algorithm (GA) in MATLAB programing environment, with RMSE function between simulated RZSM and observational values.

**Figure 7:**
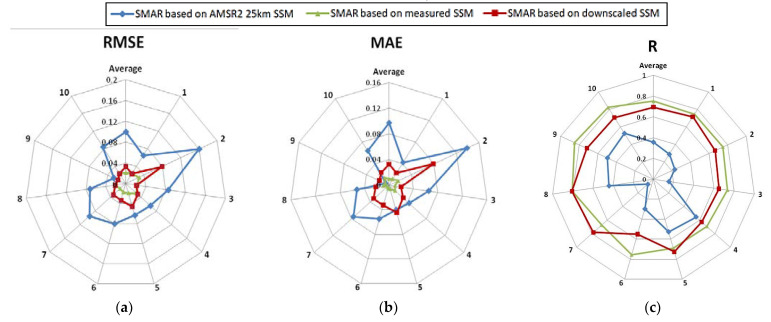
Basic statistics for the comparison results of SMAR-estimated RZSM using Schemes 1, 2, and 3 at ten stations.

Figure 7 illustrates the results of statistical analyses for the comparison results of SMAR-estimated RZSM using three schemes at ten stations. Notably, the SMAR model based on downscaled SSM has estimated RZSM with relatively high precision in most stations. The MAE and RMSE values at Station 2 were found to be higher than other stations, which could be attributed to high error values of the SSM downscaling model of this station as compared to other stations. It can, therefore, be said that in every station where the downscaling model estimates SSM at a low error rate, the SMAR model could also estimate RZSM more accurately.

**Figure 8:**
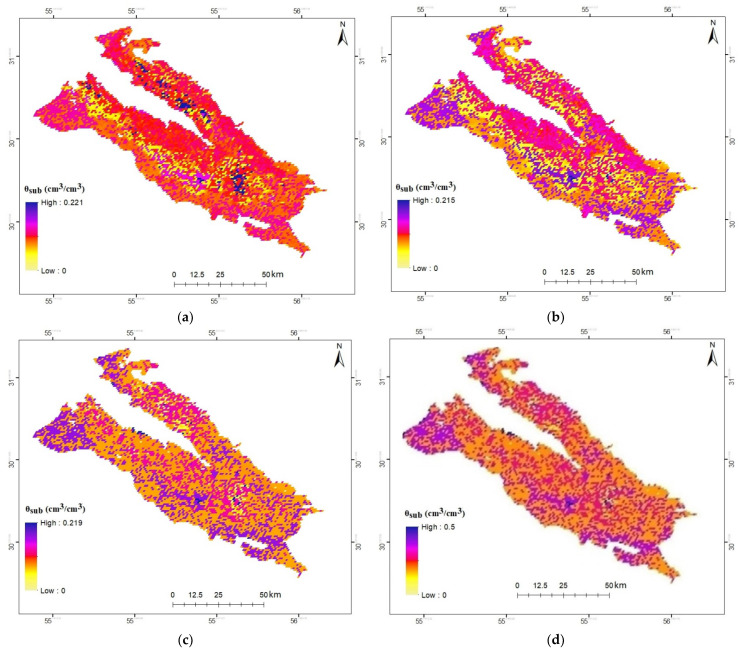
The downscaled RZSM ($\theta_{sub}$) values in Rafsanjan plain on (**a**) 9/19/2017, (**b**) 10/1/2017, (**c**) 5/31/2018, and (**d**) 8/29/2018.

The results indicated an increased accuracy of the model in estimating RZSM using the downscaled SSM data obtained from the method proposed in this study. In this method, mean R, RMSE, MAE values between all the stations were obtained equal to 0.71, 0.032, 0.032, respectively. Since SSM is considered as the most critical parameter of the SMAR model provided that it could be estimated with high accuracy, it will affect the more accurate estimation of RZSM by the SMAR model, verified by the results obtained. The downscaled SSM-based SMAR model can be utilized to develop a system for receiving downscaled SSM values to create RZSM maps. Hence, the AMSR2-downscaled SSM model was used in the SMAR model based on MODIS parameters, and the RZSM values were obtained at 1 km pixels for different days. Figure 8 depicts the downscaled RZSM sample (θ_sub_) based on the SMAR model in the Rafsanjan Plain for 29 August 2018.

## 4. Discussion

This study developed an RZSM estimation model, i.e., SMAR, at 1 km × 1 km pixels in the Rafsanjan Plain. Initially, the AMSR2 25 km SSM was compared with the ground-based SSM values in the Rafsanjan Plain. The overall validation results suggested a low correlation in most stations. In addition to the distance of the station from the midpoint of 25 km pixel, other factors such as changes in vegetation compaction, land-use, topography, rainfall, and soil properties of the study area could affect the correlation between large-scale data and measured field data. It is necessary to collect more SSM data from 25 km pixels to have stronger validation.

The coarse-resolution satellite-based SSM data necessitates the downscaling of SSM data for many hydrological and agricultural plans in the study area, contributing to a precise RZSM estimation by the SMAR model on a finer scale.

Since fine-resolution MODIS data are available globally and daily, this study considered the downscaled AMSR2 SSM based on MODIS parameters. The results demonstrated a good consistency between the downscaling method that exploits these parameters and the measured data—which were consistent with those of the previous studies [20,52]—leading to reduced error rates and increased correlation coefficients at measurement stations. The downscaled SSM was estimated using a linear equation, which correlated MODIS Albedo, LST, and NDVI. After the downscaling, at Stations 1, 2, 3, and 7, the correlation coefficients increased from 0.473, 0.400, 0.350, and 0.232 to 0.742, 0.876, 0.715, and 0.793, respectively, and the values of MAE and RMSE in the root zone decreased, indicating improved results by using the above-mentioned downscaling technique.

To obtain RZSM at 1 km pixel in the Rafsanjan Plain, the downscaled SSM model was used based on MODIS parameters in the SMAR model. RZSM obtained from the SMAR model based on downscaled SSM was compared with measurement RZSM data. The results indicated that the model could initially manage to estimate RZSM values from SSM variations, and then increase the accuracy of the model to estimate RZSM using the downscaled data obtained from the proposed method in this study. Mean R, RMSE, MAE values were obtained for all the stations equal to 0.71, 0.032, 0.032, respectively. As the most crucial parameter of the SMAR model, the accurate estimation of SSM will affect the more accurate estimation of RZSM by this model [39].

## 5. Conclusions

It is essential to estimate RZSM for different biogeochemical, ecological, and hydrometeorological applications and modeling. This study developed the SMAR model for estimating RZSM at 1 km pixels of the Rafsanjan Plain and subsequently, evaluated its variations. Spatiotemporal continuity of estimated RZSM by this model is one of the advantages of this method. SSM is the most important input parameter in the SMAR model, which will lead to a more accurate estimation of RZSM if precisely determined. Methods based on microwave remote sensing are suitable for retrieving SSM.

It is suggested to estimate other effective parameters of the model, e.g., soil moisture loss coefficient and soil texture parameters, more accurately for each pixel in the Rafsanjan Plain, optimize them with measured data, and use them in the SMAR model. It was generally concluded that the SMAR model, in which SSM was downscaled based on MODIS parameters, could accurately estimate and demonstrate the variations of the RZSM of the Rafsanjan Plain.

It is also suggested to consider other affecting variables, including rainfall that improves downscaled SSM products. Consider the effect of other VIs, including soil-adjusted vegetation index (SAVI) or simple ratio vegetation index (SRVI) on improved quality of downscaled products, and utilize the best of these indices. Optimization of downscaling approaches using sensitivity analysis (SA) by a network of sensors within the coverage area of a single AMSR2 pixel is suggested to validate the AMSR2 products and downscaled SSM values in the future studies.

## Figures and Tables

**Table 1:** Active and passive soil moisture products.

Website	Spatial Resolution (km-)	Temporal Resolution (Day)	Product Level/Version	Space Agency	Platform	Type	Sensor
https://smap.jpl.nasa.gov/ (accessed on 11 April 2018).	3 (Active), 36 (Passive)	1	L3	NASA	SMAP	Passive	SMAP
http://www.esa.int/Our_Activities/ accessed on 11 April 2018)	40	1	L2/V6	ESA	SMOS	Passive	MIRAS
https://nsidc.org/data/amsre (accessed on 11 April 2018)	25	1	L3/V2	NASA, JAXA	AQUA	Passive	AMSR-E
https://hydro1.gesdisc.eosdis.nasa.gov/ (accessed on 11 April 2018)	25	1	L3/V1	NASA, JAXA	GCOM-W1	Passive	AMSR2
https://trmm.gsfc.nasa.gov/ (accessed on 11 April 2018)	25	1	L2/V1	NASA, JAXA	TRMM	Passive	TMI
https://www.ipf.tuwien.ac.at/ (accessed on 11 April 2018)	25.5	1	L2	EUMETSAT, ESA	METOP	Active	ASCAT
https://podaac.jpl.nasa.gov/SSMI (accessed on 11 April 2018)	25	1	-	NASA	DMSP	Passive	SSM/I

**Table 2:** The SMAR parameters for different soil texture [37,51].

Soil Type	N [-]	SW [-]	SC [-]
sand	0.44	0.06	0.14
loamy sand	0.44	0.11	0.24
sandy loam	0.45	0.19	0.42
silty loam	0.50	0.27	0.57
loam	0.46	0.25	0.50
sandy clay loam	0.40	0.34	0.62
silty clay loam	0.47	0.45	0.73
clay loam	0.46	0.40	0.67
sandy clay	0.43	0.51	0.75
clay	0.48	0.56	0.80

**Table 3:** Results of linear regression modeling.

Coefficient	Value	Squared Error	T-Statistic	P (%)
a_000_	0.892618	0.0041	49.12	0.0015
a_100_	−0.93067	2.45 × 10^−5^	2.98	0.1431
a_010_	−0.00246	1.62 × 10^−5^	1.97	0.1275
a_001_	0.255988	5.31 × 10^−7^	−1.66	0.1112
a_110_	0.002259	1.02 × 10^−7^	−3.24	0.1255
a_101_	−2.13287	1.78 × 10^−9^	−0.12	0.2928
a_011_	0.0027	2.12 × 10^−9^	0.11	0.1159

**Table 4:** Basic statistics for each station for the comparison between the AMSR2 25 km SSM and downscaled SSM with ground-based SSM.

		AMSR2 25 km SSM			Downscaled AMSR2 1 km SSM		
Station	Distance (km)	MAE (m^3^/m^3^)	RMSE (m^3^/m^3^)	R (-)	MAE (m^3^/m^3^)	RMSE (m^3^/m^3^)	R (-)
1	5.84	0.028	0.030	0.473	0.012	0.015	0.742
2	8.46	0.092	0.094	0.400	0.047	0.047	0.876
3	9.73	0.043	0.044	0.350	0.011	0.013	0.715
4	2.10	0.029	0.030	0.783	0.013	0.017	0.707
5	1.97	0.027	0.029	0.707	0.025	0.029	0.782
6	2.58	0.037	0.040	0.491	0.028	0.031	0.538
7	14.27	0.050	0.051	0.232	0.026	0.032	0.793
8	2.35	0.035	0.035	0.603	0.009	0.009	0.735
9	3.46	0.004	0.004	0.666	0.002	0.004	0.802
10	1.06	0.042	0.043	0.700	0.003	0.003	0.705
Average	5.18	0.039	0.040	0.540	0.018	0.020	0.739
